# Gene Pool Subdivision of East African Sweetpotato Parental Material

**DOI:** 10.2135/cropsci2017.11.0695

**Published:** 2018-09-06

**Authors:** Maria C. David, Federico C. Diaz, Robert O. M. Mwanga, Silver Tumwegamire, Roberto C. Mansilla, Wolfgang J. Grüneberg

**Affiliations:** 1 M.C. David, F.C. Diaz, and W.J. Grüneberg, International Potato Center (CIP), P.B. 1556 Lima 12, Peru; 2 R.O.M. Mwanga, CIP, P.B. 22274, Kampala, Uganda; S. Tumwegamire, International Institute of Tropical Agriculture (IITA), P.B. 34441, Dares-Salaam, Tanzania; 3 R.C. Mansilla, Univ. Nacional Agraria La Molina, P.B. 12-056, Lima 12, Peru

## Abstract

Sweetpotato [*Ipomoea batatas* (L.) Lam] breeding is important for food security and health in East Africa (EA), and a breeding platform in Uganda provides national researchers and breeders in EA with true seed. Our objectives were to characterize genetic relationships among parental material used at the EA breeding platform. There were 135 parents and six check clones analyzed using 31 simple sequence repeat primers. An average of 7.13 alleles per primer was found, and Jaccard similarity coefficients were in the range of 0.298 to 1.00 with a mean of 0.542. Unweighted pair group cluster analysis placed most African parents in two main subclusters showing no association with morphology or geographical origin. The subclusters were also supported by principal coordinate analysis, derivative analysis of principal components, and population structure simulations. The analyzed breeding material from EA was highly genetically variable, grouped in two distinct genetic pools, and suitable to study heterosis exploiting breeding schemes.

SWEETPOTATO [*Ipomoea batatas* (L.) Lam] is a major food crop in sub-Saharan Africa (SSA). It is propagated by cloning through vine cuttings. In East Africa (EA), annual production reached 10.4 Tg in 2014 (FAOSTAT, 2018; accessed for Burundi, Ethiopia, Kenya, Rwanda, Tanzania, and Uganda). The crop is grown for food security and nutritional goals. White-and cream-fleshed storage roots are usually consumed; however, for the last two decades there has been interest in introducing orange-fleshed sweetpotato (OFSP) to alleviate vitamin A deficiency (Low et al., 2001). Additionally, sweetpotato leaves are used as a vegetable, and aboveground biomass has become important as animal feed in the past decade (Low et al., 2009). Efficient sweetpotato breeding programs are important to increase food security and improve health in SSA, and a basic aspect of breeding is the characterization of crossing parents.

Varietal development is straightforward in sweetpotato because each true seed plant already represents a potential variety. Selection can be accelerated by reducing test years and increasing the number of locations. Population improvement is challenging because the crop is a highly heterozygous hexaploid hybrid. This nurtures the expectation that heterosis is important for sweetpotato performance, and that systematic exploitation of heterosis can improve the efficiency of population improvement (Grüneberg et al., 2015). True seed production occurs easily by open pollination, and a successful pollination results in one to four true seeds, so polycrosses have been the backbone of population improvement (Martin and Jones, 1986). Breeding in EA started in Rwanda and Uganda in 1983 and 1987, respectively, on the basis of polycrosses with 15 to 30 parents. In 2003, CIP and HarvestPlus (Pfeiffer and McClafferty, 2007) started an initiative to breed OFSP in SSA. Within this, National Agriculture Research Systems in Tanzania, Kenya, and Ethiopia started to breed sweetpotato. In 2008, the program at Namulonge in Uganda considerably increased the number of parents (increased to 140–150 parents) and became a CIP breeding platform for EA serving National Agriculture Research Systems with true seed supply, technical backstopping, and support in proposal writing. Since 2009, this platform has been supported by the Bill & Melinda Gates Foundation. The major traits of interest in EA are: (i) resistance to sweetpotato virus disease (SPVD, interaction of *Sweetpotato chlorotic stunt virus and Sweetpotato feathery mottle virus*; farmers usually propagate sweetpotato by cloning, without obtaining new seeds regularly), (ii) storage root yields and the number of commercial storage roots per plant, (iii) elevated pro-vitamin A with high root dry matter and starchy taste, (iv) abundant upper biomass production to facilitate vine production for seed systems and for use as animal feed, and (v) earliness and suitability for piece-meal harvest. For further details on breeding targets and an outline of seed systems in EA in linkage with new variety releases, refer to Mwanga et al. (2017).

Theoretically, population improvement by polycross breeding nurseries should be inferior to controlled cross breeding. However, polycrosses of sweetpotato can easily generate large amounts of true seed. In contrast, controlled crosses allow selecting of parents on the basis of offspring performance and to test and perhaps implement a heterosis exploiting breeding scheme (HEBS). The prerequisite for a HEBS is searching for or developing mutually heterotic gene pools in parental material (Melchinger, 1999). Moreover, a HEBS also allows enhanced inbreeding within gene pools, and this is desirable for sweetpotato breeding in EA because of resistance to SPVD, which is thought to be recessively inherited and occurs at very low frequencies (Mwanga et al., 2002a, 2002b). The pressure of SPVD is extremely high in EA. An intensive discussion has commenced concerning the use of HEBS in clonal breeding of the CGIAR programs (Miles, 2007; Grüne-berg et al., 2009). Use of HEBS in clonal breeding was proposed long ago (Hull, 1945; Melchinger and Gumber, 1998), but recommendations were applied to heterosis in traditional hybrid crops and not to population improvement of the Root, Tuber and Banana program in the CGIAR. Sugarbeet (*Beta vulgaris* L.) is an exception concerning use of HEBS in root crops (Bosemark, 2006), but this crop is propagated by true seed. There are attempts to use HEBS in potato (*Solanum tuberosum* L.) breeding (Lindhout et al., 2011; Jansky et al., 2016), but these are in experimental stages and have not been adopted by breeders. We hypothesize that the large number of sweetpotato parents in the crossing block at Namulonge in Uganda can be grouped into gene pools to serve as a basis to test and implement HEBS for sweetpotato breeding in EA.

The establishment of heterotic groups can be based on geographic origin, agronomic traits, pedigree data, or molecular data (Melchinger, 1999). Mutually heterotic gene pools can be developed in sweetpotato, as has been shown for two South American OFSP breeding populations, and simple sequence repeat (SSR) markers were very useful in this context (Federico Diaz, personal communication, 2014; results partially described in Grüneberg et al., 2015). Molecular markers have been demonstrated to be a useful tool to group parental material into gene pools for HEBS in many crops: maize (*Zea mays* L.; Messmer et al., 1992; Reif et al., 2005), rye (*Secale cereale* L.; Fischer et al., 2010), winter triticale (× *Trticosecale* Wittm.; Tams et al., 2004), oilseed rape (*Brassica napus* L.; Becker et al., 1995), faba bean (*Vicia faba* L.; Link et al., 1995), rice (*Oryza sativa* L.; Xiao et al., 1996) and sorghum [*Sorghum bicolor* (L.) Moench; Bhosale et al., 2011]. African sweet-potato germplasm has been repeatedly characterized as having high morphological diversity in EA (Kaledzi et al., 2010; Yada et al., 2010a; Elameen et al., 2011). Agronomic traits indicated high diversity in local EA farmer varieties (FVs) (Tumwegamire et al., 2011a).

With respect to molecular markers, sweetpotato germplasm from SSA has been investigated using amplified fragment length polymorphism (Elameen et al., 2008) and SSR markers (Gichuru et al., 2006; Yada et al., 2010b; Tumwegamire et al., 2011b). These studies predominantly used FVs and not the entire parental material of a breeding program. Analysis of FVs from EA (*N* = 266) with four microsatellite markers showed no relationships between geographic origin and clustering of clones (Gichuru et al., 2006). For 94 Tanzanian cultivars analyzed using amplified fragment length polymorphism markers, there were no correlations between morphological and molecular data (Elameen et al., 2008). High genetic diversity was detected for 192 Ugandan germplasm accessions characterized by 10 microsatellite markers, but there was no relationship between the district of clone origin and marker clustering (Yada et al., 2010b). In a study using 26 microsatellite markers to compare white-fleshed and rarely found OFSP FVs from SSA, the OFSP from Africa was clearly distinct from OFSP from the Americas and closely related to white-fleshed sweetpotato from Africa (Tumwegamire et al., 2011b). Parental material used in EA breeding nurseries has not been studied using molecular markers for gene pool subdivision.

This study had two objectives. The first was to characterize genetic relationships among sweetpotato parents with microsatellite markers used by CIP’s breeding platform in EA at the National Crops Resources Research Institute at Namulonge in Uganda. The second objective was to allocate parents of the Namulonge polycross crossing block into two potentially heterotic gene pools and to identify potential cross combinations for further studies on HEBS.

## MATERIALS AND METHODS

### Plant Material

A total of 141 sweetpotato accessions were used for this study (Table 1) and described by clone name, local code, cultivar type, country of origin, plant type, root flesh and skin color, storage root form, and CIP and Ugandan germplasm codes (Supplemental Table S1). Of these, 135 accessions were parents from the polycross crossing block at Namulonge in Uganda, and six clones from the CIP genebank in Peru were used as checks. The parents were mainly of EA origin: 119 from EA, one from South Africa, two from West Africa, and 13 of non-African origin. Parents mainly comprised FVs (108 clones) and some modern varieties (MVs) or breeding lines (27 clones); 28 parents were OFSP, and 107 parents were non-OFSP. Check clones (CCs) were not part of the polycross recombination and included ‘Jonathan’ (FV from Peru), ‘Xushu 18’ and ‘Yanshu 1’ (MVs from China), ‘Naveto’ (FV from Papua New Guinea), ‘Resisto’ (MV from the United States), and ‘SPK004’ (FV from Kenya held in trust at CIP’s genebank in Peru). All CCs were obtained as plantlets from CIP’s genebank, whereas all parental material from the crossing block was obtained as lyophilized leaves from Uganda.

**Table 1:** Description of clones used for the genetic diversity study.

Origin	No. of clones	Description by clone name, code, country of origin, variety type, and storage root flesh color
Parental material of East African origin	119	Clone name and code: K-118 (KE09), Oguroiwe (KE11), K-566632 (KE14), SPK004 (KE19), Ubuogo (KE21), Mugande RW01), Carrot Dar (TZ01), Mayai (TZ02), Carrot C (TZ03), Ukerewe (TZ04), Magabari (UG05), NN (UG06), Karebe UG15), Kigabali (UG19), Kyebandula (UG20), Tororo 3 (UG23), Osapat (UG29), Kala (UG40), Abuket 1 (UG41), Ejumula (UG43), Kamamanzi (UG44), NN (UG45), Wagabolige (UG47), Osukut (UG51), Opaade (UG52), Kakoba UG53), Epura Amojong (UG54), Anyumel (UG55), Oleke (UG56), Kalobo (UG57), Rwabuganda (UG58), Kibogo UG59), NK318L (UG60), NN (UG61), Kyebandira 2 (UG62), Anamoyito (UG63), Mary (UG64), Silk Omuyaka (UG65), Koromojo (UG66), Tedolo Kereni (UG67), Liralira (UG68), Kahungezi (UG69), Burundi (UG70), NN (UG71), Kalebe UG72), Kibanda (UG73), NN (UG74), Bunduguza Empyaka (UG75), Dimbuka Obuleku (UG76), Dimbuka (UG77), Silk UG78), Otada (UG79), NASPOT 1 (UG80), NASPOT 5 (UG81), Kyabafuluki (UG82), NASPOT 5/58 (UG83), Kampala Red (UG84), Silk (UG85), NASPOT 3 (UG86), NN (UG87), Dares-Salaam Carrot (UG88), Suwedi (UG89), NN (UG90), Tuulansime (UG91), Bungoma (UG92), Duduma 2 (UG93), NN (UG94), Dduka Enzala (UG95), Koromojo Red (UG96), Bikiramaia (UG97), NN (UG98), NN (UG99), NN (UG100), Oketodede (UG101), Nylon (UG102), NN (UG103), Uganda Mali (UG104), NN (UG105), Munafu Dimbuka (UG106), NN (UG107), Mugiga (UG108), Bunduguza 2 (UG109), Kigaire UG110), Namusoga (UG111), NN (UG112), Koromojo Red (UG113), Bunduguz Empyaka 2 (UG114), Gulu (UG115), Dimbuka (UG116), New Kawogo (UG117), Bitambi (UG118), Sowola 389A (UG119), NN (UG120), NASPOT 7 (UG121), NASPOT 10 O (UG123), NASPOT 11 (UG124), NK259L (UG125), Semanda (UG126), NN (UG127), Mukoma (UG128), Silimu (UG129), NN (UG130), NN (UG131), NN (UG132), Dimbuka-Bukulula (UG133), Dagadaga (UG134), Kawogo Old (UG135), NN (UG136), Woluganda (UG137), BND145L (UG138), Tengerere (UG139), Mpaifumbiro (UG140), Mpambire (UG141), Tanzania (UG142), Silk (UG143), Malagalia (UG144), Mbale (UG145), and Tanzania (UG146). Country of origin: Kenya, Rwanda, Tanzania and Uganda with a frequency of 5, 1, 5 and 108, respectively. Variety type: breeding line, FV, and MV† with a frequency of 4, 105, and 10, respectively. Storage root fresh color: white, cream, pale yellow, yellow, pale orange, orange, and dark orange with a frequency of 14, 68, 1, 18, 4, 9, and 4, respectively.
Parental material of Southern African origin	1	Clone name and code: Rainha (MZ01). Country of origin: Mozambique. Variety type: FV. Storage root fresh color: pale yellow.
Parental material of West African origin	2	Clone name and code: TIS-9265 (CIP440076) and TIS-9101 (CIP440099). Country of origin: Nigeria. Variety type: MV. Storage root fresh color: cream and orange.
Parental material of non-African origin	13	Clone name and code: Santo Amaro (CIP400011), Dagga (CIP199062.1), Huarmeyano (CIP420020), Zapallo (CIP420027), Tainung 64 (CIP440189), Beauregard (CIP440132), Caromex (CIP440136), DLP3163 (CIP420269), Excel (CIP440016), Jewel (CIP440031), Resisto (UG), W-115 (CIP440424), and WT-237 (n.a.‡). Country of origin: Brazil, Peru, Taiwan, USA, and n.a. with a frequency of 1, 4, 1, 6, and 1, respectively. Variety type: FV and MV with a frequency of 2 and 11, respectively. Storage root fresh color: cream, yellow, orange, and dark orange with a frequency of 1, 2, 8, and 2, respectively.
Check clones	6	Clone name and code: Xushu 18 (CIP440025), Yanshu 1 (CIP440024), SPK004 (CIP441768), Naveto (CIP440131), Jonathan (CIP420014), and Resisto (CIP440001). Country of origin: China, Kenya, Papua New Guinea, Peru, and USA with a frequency of 2, 1, 1, 1, and 1, respectively. Variety type: FV and MV with a frequency of 2 and 4, respectively. Storage root fresh color: white, cream, pale yellow, orange, and dark orange with a frequency of 1, 2, 1, 1, and 1, respectively.

† FV, farmer variety; MV, modern variety.

‡ n.a., not available.

### SSR Amplification

Lyophilized leaves were used to isolate total DNA by the method of Murray and Thompson (1980). DNA samples were quantified, and 60 ng of total genomic DNA from each sample was used per polymerase chain reaction (PCR). All reactions had a total volume of 10 μL containing 2.5 mM MgCl2, 0.2 mM deoxynucleotides, 22 nM forward primer, 15 nM reverse primer, 25 nM M13 Forward 700/800 (LI-COR), and 0.025 U μL^−1^ *Taq* DNA polymerase (New England Biolabs), each as a final concentration. The thermocycler amplification program had a first step at 94°C that lasted 4 min, then 29 cycles as follows: denaturing at 94°C for 1 min, alignment for 1 min, and extension at 72°C for 1 min. There was a final of extension step of 72°C for 7 min, and the temperature was then dropped to 4°C. Annealing temperatures for PCR varied according to the primer (Table 2). The PCR products were run in a poly-acrylamide gel in a DNA Analyzer 4000 (LI-COR).

**Table 2:** Description of simple sequence repeat markers used to characterize sweetpotato genotypes used at the breeding platform in Uganda by currently used names, motifs, forward and reverse primers, and annealing temperature.

Name	Forward primers	Reverse primers	Motif	Temp.	Reference
	————————— 5ʹ–3ʹ —————			°C	
Ib-242	GCGGAACGGACGAGAAAA	ATGGCAGAGTGAAAATGGAACA	(CT)_3_CA(CT)_11_	58.0	Buteler et al. (1999)
Ib-286	AGCCACTCCAACAGCACATA	GGTTTCCCAATCAGCAATTC	(CT)_12_	57.0	Buteler et al. (1999)
Ib-297	GCAATTTCACACACAAACACG	CCCTTCTTCCACCACTTTCA	(CT)_13_	58.0	Buteler et al. (1999)
IBCIP-1	CCCACCCTTCATTCCATTACT	GAACAACAACAAAAGGTAGAGCAG	(ACC)_7_	63.0	Yañez (2002)
IbC5	CCACAAAAATCCCAGTCAACA	AGTGGTCGTCGACGTAGGTT	(AAG)_8_	62.0	Solis et al. (unpublished data, 2008)
IbC12	TCTGAGCTTCTCAAACATGAAA	TGAGAATTCCTGGCAACCAT	(TTC)_6_	56.0	Solis et al. (unpublished data, 2008)
IbE2	CAGCCGCCAAGTTTTCTACA	AGGCGGAGGCTGATAATGA	(TCT)_13_	62.0	Solis et al. (unpublished data, 2008)
IbJ67	CACCCATTTGATCATCTCAACC	GGCTCTGAGCTTCCATTGTTAG	(GAA)_5_	58.0	Solis et al. (unpublished data, 2008)
IbJ116A	TCTTTTGCATCAAAGAAATCCA	CCTCAGCTTCTGGGAAACAG	(GAA)_8_	57.0	Solis et al. (unpublished data, 2008)
IbJ263	CTCTGCTTCTCCTGCTGCTT	GTGCGGCACTTGTCTTTGATA	(AAC)_5_	55.5	Solis et al. (unpublished data, 2008)
IbJ522a	ACCCGCATAGACACTCACCT	TGACCGAAGTGTATCTAGTGG	(CAC)_6–7_	57.0	Solis et al. (unpublished data, 2008)
IbJ544b	AGCAGTTGAGGAAAGCAAGG	CAGGATTTACAGCCCCAGAA	(TCT)_5_	62.0	Solis et al. (unpublished data, 2008)
IbJ664E	CACATGCCATGGACGCTCCAA	GATTCTTCTCCTTCCAGCTCCT	(CTT)_6_	55.0	Solis et al. (unpublished data, 2008)
IbN21	AACCCTCATCTTTCTCATCTCTTC	ACCTTGAACTCCGTCTCCTCTT	(CT)_0_C	60.0	Huamani et al. (unpublished data, 2010)
IbN24	TAATGAGGTGTGATGATGGGTACTA	AGTGAAGTTGAGGTCAGGAAAATC	(TA)_5_GA(TA)_3_	60.0	Huamani et al. (unpublished data, 2010)
IbN37	GATGATGGAGCTCATAAATCTCG	GTCACTGTGTCCTCCAGTTTTTC	(TA)_7_T	55.0	Huamani et al. (unpublished data, 2010)
IBS144	TCGAACGCTTTCTACACTCTT	CTGTGTTTATAGTCTCTGGCGA	(TTC)_9_	60.0	Schafleitner et al. (2010)
IBS147	TGTGTACATGAGTTTGGTTGTG	GAAGTGCAACTAGGAAACATGA	(GCA)_8_	55.0	Schafleitner et al. (2010)
IBS149	CCACCTCCTTAGGTATCAGACT	ACTACTAGCGCTGCAACCTTAT	(AGA)_8_	60.0	Schafleitner et al. (2010)
IBS169	CGTACTATGTTTCCCCCATTAC	AATGCATCTACCCTCCTTACAC	(TTG)_8_	53.0	Schafleitner et al. (2010)
IBS199	TAACTAGGTTGCAGTGGTTTGT	ATAGGTCCATATACAATGCCAG	(ACA)_7_	60.0	Schafleitner et al. (2010)
IbY40	AGTGTTGGGACTCATAAAGATTCTG	GAATGAAATACAGTGACCCGAGAG	(GCG)_7_GC	60.0	Huamani et al. (unpublished data, 2010)
IbY44	CAAGAAGAGCATAAGCGTGAGAT	GCGATCTGAGAAGGTGATAATTG	(AGA)_6_	52.0	Huamani et al. (unpublished data, 2010)
IbY46	TAGTAACACCATTACTTATTAACTTTG	TGTAATCTCATGGATTGCTCGTAG	(ATC)_5_AT	55.0	Huamani et al. (unpublished data, 2010)
IbY51	GATGTCGTTTAGCGGACTGAG	GTATCGTCACATTCAGCAGCAG	(GCG)_5_G	55.0	Huamani et al. (unpublished data, 2010)
IbY52	AAACAGATAGCAGAGACGAGATGAG	CAGATAGTGTCACCAACACTGAAGA	(GCG)_5_G	55.0	Huamani et al. (unpublished data, 2010)
IbY53	CCACGATCTCGGAAACCGCCAT	GGGGCAAAAGGTCTTATTCATAT	(GGA)_5_G	55.0	Huamani et al. (unpublished data, 2010)
IbY54	GTCCAAGAGAAAGAAACTGAAGATG	AACTATTCTGCACAACTACATGCTC	(TGT)_5_T	57.0	Huamani et al. (unpublished data, 2010)
IbY56	CACCATGGATTTCAAACCACTACTT	AGGGGGAGTTGTCTTGACTGGT	(CCT)_5_	52.0	Huamani et al. (unpublished data, 2010)
IbY58	ACGACATGGCTCTCTCTTTCTC	AGTTTCCTTTCTCGACGCTTCT	(GCG)_5_	55.0	Huamani et al. (unpublished data, 2010)
IbY60	TCTCTCTGTTATGTTATGGTGATGG	GCGTTTTACAAGATTCAGAAACCAC	(TAT)_5_	62.0	Huamani et al. (unpublished data, 2010)

### SSR Data Scoring and Analysis

Genotypes were scored for the presence (1) or absence (0) of bands visualized using the software Saga^GT^ version 3.3. (LI-COR, 2005). The number of alleles per locus, percentage of polymorphic loci, and polymorphic information content (PIC) were calculated. The PIC was calculated with the equation

$$PIC=1-\sum_{i=1}^{n}p_{i}^{2}$$

where *p_i_* is the frequency of the *i*th allele (Weir, 1996). The limit of the discriminating power (*D*_L_) was calculated with the equation

$$D_{L}=1-\sum_{i=1}^{n}p_{i}^{2}$$

where *p_i_* is the frequency of the *i*th haplotype (Kloosterman et al., 1993).

A similarity matrix was obtained by the Jaccard similarity coefficient and a dendrogram with the unweighted pair group method analysis (UPGMA) using the software NTSYSpc version 2.2 (Rohlf, 2000). On the basis of the UPGMA results, a subdataset was created comprising parents of African origin with potential to be grouped into two gene pools (i.e., presence and absence data of 119 genotypes). On the subdataset, a principal coordinates analysis (PCoA) was performed using DARwin version 6 (Perrier and Jacquemoud-Collet, 2006). Next, the subdataset was recoded as alleles as requested by the “adegenet” package ( Jombart, 2008) and artificial alleles were eliminated using the “poppr” package (Kamvar et al., 2014) to allow a discriminant analysis of principal components (DAPC) using “adegenet” in the software R (R Core Team, 2014). For the DAPC, four clusters were chosen because they had the lowest Bayesian information criterion, and 30 principal components (PCs) were retained. To corroborate gene pool allocation of genotypes, a simulation of population structure was performed with Structure software (Pritchard et al., 2000) for *K*-values of 1 to 10 with the following parameters: burn-in period of 100,000, 100,000 Markov chain Monte Carlo repetitions, and 100 runs. Results were analyzed as described by Evanno et al. (2005), and Δ*K* was plotted with *K*-values of 1 to 10, where Δ*K* is the mean of the second-order rate of change of the ln*P*(*D*) values of a given *K* divided by the ln*P*(*D*) SD (Evanno et al, 2005). Finally, an analysis of molecular variance (AMOVA) was performed using Arlequin 3.5.2.2 software (Excoffier et al., 2005) with marker data of the parents ascribed to the two proposed gene pools to divide the genetic variation into components attributed to the variance within and between proposed pools after excluding SPK004 (from the CIP genebank), a CC that was not part of the parent material at Namulonge.

## RESULTS

The 31 SSR markers used (Table 2) proved suitable for analyzing the genetic diversity of sweetpotato parents used in the EA breeding platform. A total of 221 alleles were obtained: seven were monomorphic, and 214 were polymorphic (Table 3). On average, 7.13 alleles per locus were obtained. The number of alleles per SSR marker locus ranged from 2 to 12. The average PIC was 0.75. The *D*_L_ was in the range of 0.26 to 0.97, with an average of 0.82. For 14 primer pairs, *D*_L_ was >0.90.

**Table 3:** Number of alleles, polymorphic information content (PIC), and power of discrimination (*D*_L_) for 31 simple sequence repeat loci used to estimate the genetic diversity in parental material from Uganda and checks.

Primer name	No. of alleles	PIC	*D*_L_
Ib-242	5	0.76	0.76
Ib-286	8	0.83	0.95
Ib-297	9	0.82	0.96
IBCIP-1	4	0.75	0.74
IbC5	8	0.79	0.91
IbC12	7	0.84	0.96
IbE2	12	0.8	0.93
IbJ67	8	0.78	0.89
IbJ116a	9	0.81	0.93
IbJ263	5	0.72	0.82
IbJ522a	5	0.75	0.82
IbJ544b	4	0.52	0.26
IbJ664E	4	0.59	0.46
IbN21	9	0.81	0.90
IbN24	4	0.71	0.69
IbN37	12	0.85	0.97
IBS144	9	0.84	0.96
IBS147	9	0.82	0.94
IBS149	12	0.8	0.93
IBS169	5	0.68	0.76
IBS199	11	0.84	0.95
IbY40	7	0.76	0.92
IbY44	8	0.78	0.89
IbY46	8	0.82	0.94
IbY51	6	0.69	0.61
IbY52	6	0.77	0.86
IbY53	5	0.72	0.76
IbY54	6	0.69	0.76
IbY56	2	0.48	0.47
IbY58	5	0.74	0.88
IbY60	9	0.76	0.89
Average	7.13	0.75	0.82

**Fig. 1:**
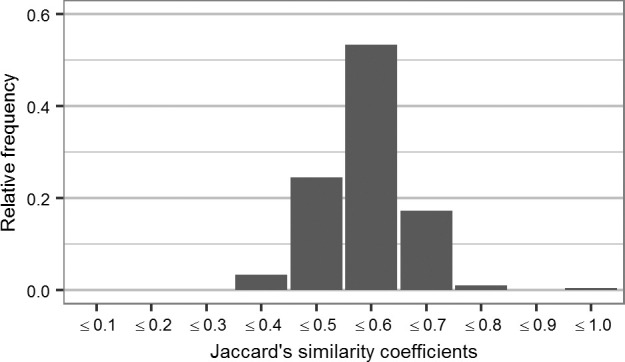
Frequency distribution of Jaccard genetic similarity distances among 141 sweetpotato genotypes (135 parents from the East African breeding platform and six check clones).

**Fig. 2:**
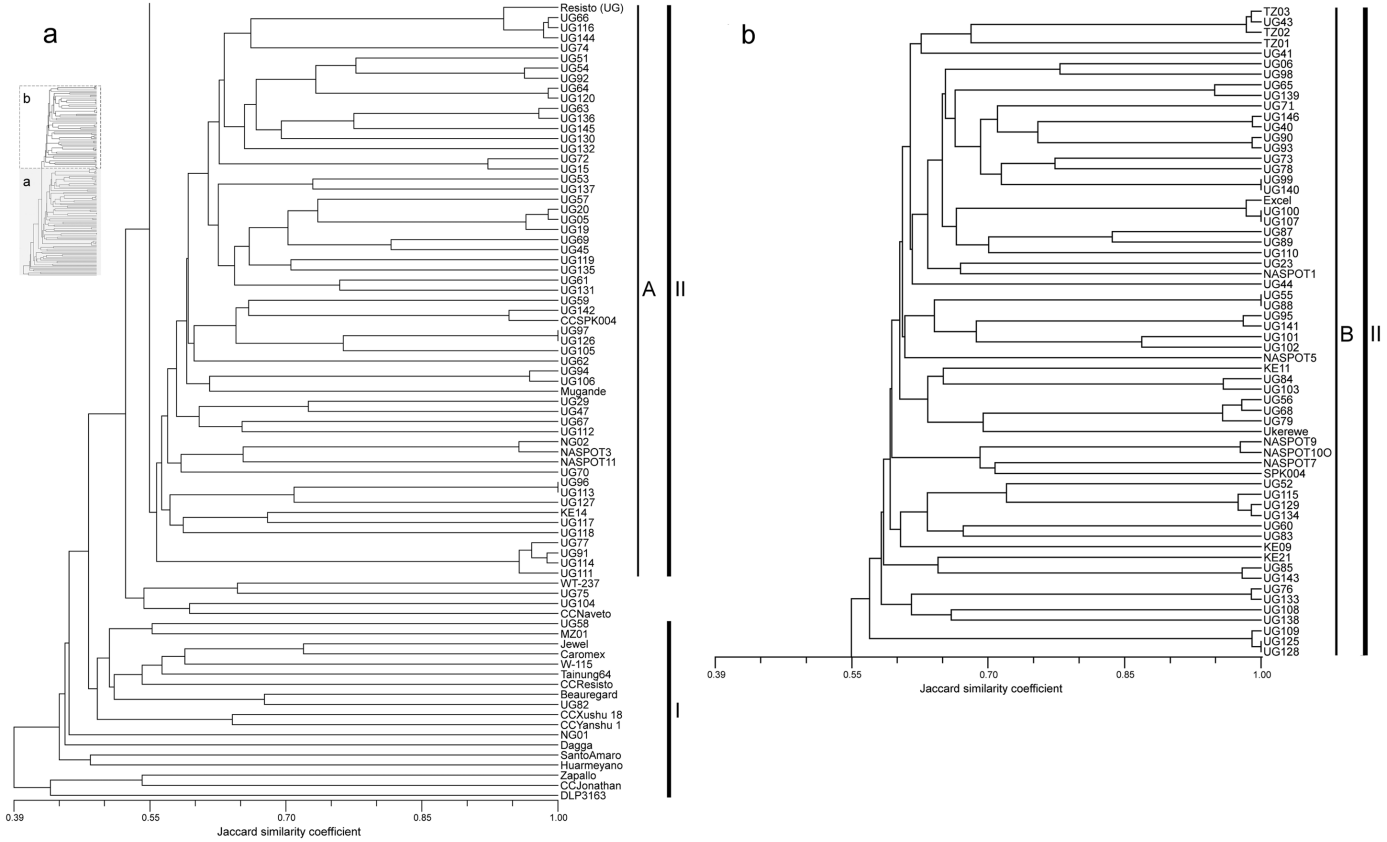
Dendrogram of the unweighted pair group method analysis based on Jaccard’s genetic similarity distances for simple sequence repeats of 135 parents in use at the East African breeding platform and six check clones (labeled by check clone [CC] and variety name).

The Jaccard similarity coefficient and the dendrogram generated with UPGMA revealed large differences among genotypes (Fig. 1 and 2). Jaccard coefficients were in the range of 0.298 to 1.000 (Fig. 1). There was relatively low similarity among most genotypes, with a mean of 0.542 across all pairwise distances. The dendrogram generated (Fig. 2) grouped non-African and African accessions separately (Clusters I and II, respectively). Non-African material clustered according to geographic origin. Four out of six CCs were in Cluster I, whereas CC SPK004 (origin Kenya, from CIP genebank) was in Cluster II and CC Naveto from Papua New Guinea was in an independent subcluster (neither Cluster I nor II), together with clone ‘WT-237’ of unknown origin and two African genotypes. The non-African accessions in Cluster I started to merge with African accessions (Cluster II) at a Jaccard coefficient of 0.49. The MVs ‘TIS-9265’ (NG01) and ‘Dagga’ (199062.1) from breeding of IITA and CIP, respectively, were classed in Cluster I but were not part of any subcluster group. The MVs from China (Xushu 18 and Yanshu 1) grouped at a Jaccard coefficient of 0.49, but the MV ‘Tainung 64’ from Taiwan clustered with US varieties. The MVs from the United States—‘Jewel’, ‘Caromex’, ‘W-115’, CC Resisto (from the CIP genebank), and ‘Beauregard’—grouped consistently. The CC Resisto from the CIP genebank (Cluster I) was very different from the genotype named Resisto (UG) used as a parent in the EA breeding platform (Cluster II).

Most parents from EA (117 clones) grouped in Cluster II (Fig. 2). There was no relationship between grouping of clones and country of origin or district of collection (Table 1, Supplemental Table S1). Six accession pairs in Cluster II were respectively similar (Fig. 2, Supplemental Table S2). At a higher aggregation level, there were two main subclusters in Cluster II at similarity 0.54 (designated Groups A and B in Fig. 2). In Group A of Cluster II (comprising 57 accessions), all genotypes were of EA origin (Fig. 2a). Some smaller subclusters appeared in Group A; for example, at a Jaccard coefficient of 0.95 comprising ‘Dimbuka’ (UG77), ‘Tuulansime’ (UG91), ‘Namusoga’ (UG111), and ‘Bunduguza Empyaka 2’ (UG114), all clones had white-to cream-fleshed root color and pink or purple-red storage root skin color (Supplemental Table S1). Most genotypes in Group A were FVs from different districts of Uganda or different EA countries, and no morphological characteristic distinguished the group. The MVs TIS9101 (NG02) and ‘NASPOT 3’ (UG86) morphologically differed despite their genetic similarity (Supplemental Table S1). The CC SPK004 (from the CIP genebank) and Resisto (UG, a parent in the EA breeding platform) both fell in Group A of Cluster II.

On the basis of SSR marker data, flesh color and storage root shape, the CC SPK004 differed from the SPK004 used as a parent (Group B of Cluster II). Resisto (UG) clustered at high similarity with African clones. Although its orange flesh root color was very close to CC Resisto, on the basis of SSR marker data, plant type, storage root shape, and skin color, Resisto (UG) clearly differed from CC Resisto (Supplemental Table S1). In Group B of Cluster II (comprising 62 accessions), all genotypes were of EA origin, except ‘Excel’ (from the United States). For 11 pairs of genotypes in Group B, Jaccard coefficients were 0.90 to 1.00 (Supplemental Table S2). As for Group A of Cluster II, these pairs in Group B of Cluster II were not geographically associated. All OFSP FVs were found in Group B. Two subclusters in Group B contained OFSP exclusively: the first subcluster comprised Carrot D (TZ01), ‘Mayai’ (TZ02), ‘Carrot C’ (TZ03), ‘Abuket 1’ (UG41), and ‘Ejumula’ (UG43); and the second comprised SPK004 (KE19) and MVs derived from SPK004 (KE19) such as ‘NASPOT 7’ (UG121),’ NASPOT 9 O’ (UG122), and ‘NASPOT 10 O’ (UG123), all of which are moderately resistant to SPVD.

**Fig. 3:**
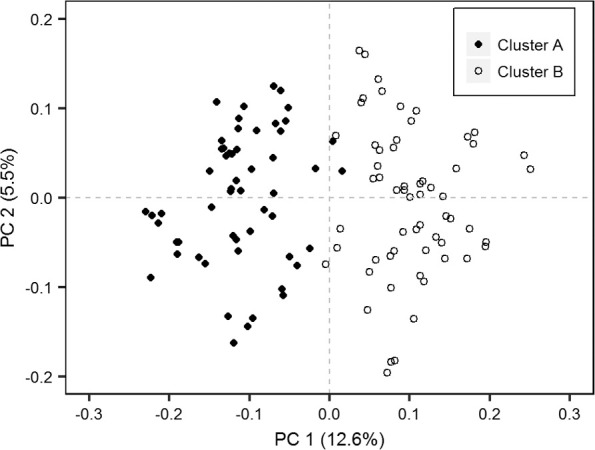
Principal coordinate analysis based on Jaccard’s genetic similarity distances for simple sequence repeats of 118 sweetpotato genotypes from Cluster II and in use as parents at the East African breeding platform (Groups A and B in Fig. 2, except for check clone CC-SPK004, designated as Cluster A and B). PC1 and PC2 are the first and second principal coordinates, respectively.

**Fig. 4:**
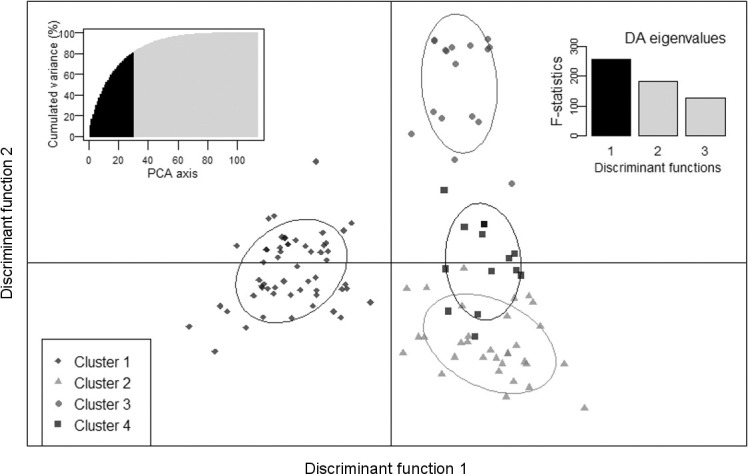
Discriminant analysis of principal components (DAPC) based on Jaccard’s genetic similarity distances for simple sequence repeats of 118 sweetpotato genotypes from Cluster II and in use as parents at the East African breeding platform (Groups A and B in Fig. 2, except for check clone CC-SPK004). The left upper inset graphic shows the cumulative variance by principal component analysis (PCA) axis retained; the right upper inset shows the discriminant analysis (DA) eigenvalues; the left lower insert shows the symbols used for genotypes allocated to DAPC Clusters 1 to 4.

**Fig. 5:**
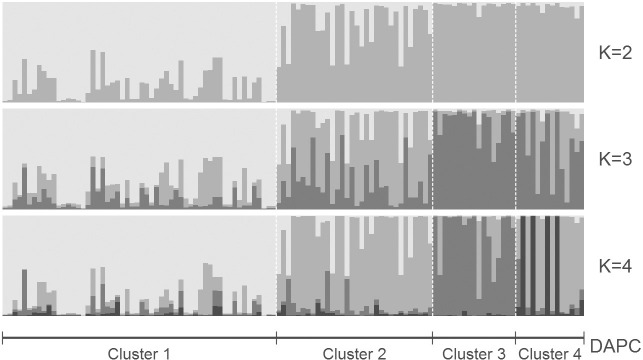
Bayesian model-based inference of simulated population structure on 118 sweetpotato genotypes from Cluster II and in use as parents at the East African breeding platform (Groups A and B in Fig. 2, except for check clone SPK004). Simulations presented are for *K*-values of 2 to 4.

In further analysis steps, all binary SSR data belonging to genotypes of Cluster I were excluded from the analysis to determine whether Cluster II could be separated into two or more groups (as UPGMA indicated). This was performed in four analysis steps: (i) PCoA and a biplot of PC score values, (ii) DAPC, (iii) a simulation of population structure, and (iv) AMOVA. The first two PCs for Cluster II genotypes explained 18.1% of the total variation. The biplot of the first two PC axis score values (Fig. 3) separated Groups A and B of Cluster II (Fig. 2). The DAPC separated the accessions into four clusters and explained 80.9% of the variance. The first component separated DAPC Cluster 1 from DAPC Clusters 2 to 4 (Fig. 4). The DAPC Cluster 1 contained the accessions of Group B in Cluster II (Fig. 2b), except for seven clones observed in DAPC Cluster 2. All remaining genotypes in DAPC Clusters 2 to 4 were found in Group A in Cluster II (Fig. 2a), except for ‘Osukut’ (UG51). The DAPC Cluster 3 contained 15 of 17 genotypes found in Group A of Cluster II at the top of the dendrogram (Fig. 2a). The DAPC Cluster 4 contained genotypes from two groups at the bottom of Group A of Cluster II. Simulation of the population structure using Structure (Pritchard et al., 2000) confirmed the PCoA and DAPC results. The Δ*K* obtained for *K*-values of 1 to 10 exhibited a clear peak at *K* = 2 (results not presented). The simulations were plotted for *K*-values of 2 to 4 (Fig. 5). For *K* = 2, two groups were well differentiated: the first included genotypes of DAPC Cluster 1, and the second included genotypes from DAPC Clusters 2 to 4. For *K* = 3, the newly appearing group contained mostly genotypes from DAPC Clusters 3 and 4—this new group in the simulated population structure was represented by two groups within Group A of Cluster II (Fig. 2a). The first group located at a Jaccard coefficient of 0.6 contained 29 accessions, and the second group contained four clones joining at a Jaccard coefficient of 0.94. For *K* = 4, similarities between DAPC Clusters 2 and 4 increased. For simulations with *K* > 4, the genotypes were allocated in the same pattern, but genotypes from DAPC Cluster 1 started to differentiate into new groups (results not shown).

With respect to parental material with African origin at Namulonge, two gene pools were proposed (Table 4). The first comprised Group A of Cluster II genotypes (57 clones), and the second comprised Group B of Cluster II genotypes (62 clones) (Fig. 2). Only six African clones from the crossing block remained unclear concerning gene pool allocation (Fig. 2): ‘Bunduguza’ (UG75) and ‘Uganda Mali’ (UG104) in the independent subcluster (neither Cluster I nor II); and ‘Rwabuganda’ (UG58), ‘Kyabafuluki’ (UG82), TIS-9265 (NG01), and ‘Rainha’ (MZ01) in Cluster I together with exotic parental material for EA or CCs from North and South America and Asia. For the proposed gene pool allocation, AMOVA revealed 14.0% variation between and 85.0% variation within pools (results not presented; CC SPK004 was excluded from AMOVA because it was not used as a parent at Namulonge). The variation between gene pools was significant (*P* < 0.0001).

**Table 4:** Proposed grouping of parental material in the East African breeding platform.

Group	No. of genotypes	Clone names (codes)
Group A, Cluster II	57 (56†)	K-566632 (KE14), Mugande (RW01), Magabari (UG05), Karebe (UG15), Kigabali (UG19), Kyebandula (UG20), Osapat (UG29), NN (UG45), Wagabolige (UG47), Osukut (UG51), Kakoba (UG53), Epura Amojong (UG54), Kalobo (UG57), Kibogo (UG59), NN (UG61), Kyebandira 2 (UG62), Anamoyito (UG63), Mary (UG64), Koromojo (UG66), (UG67), Tedolo Kereni (UG69), Burundi (UG70), Kalebe (UG72), NN (UG74), Dimbuka (UG77), NASPOT 3 (UG86), Tuulansime (UG91), Bungoma (UG92), NN (UG94), Koromojo Red (UG96) and Koromojo Red (UG113)†‡, Bikiramaia (UG97) & Semanda (UG126)‡, NN (UG105), Munafu Dimbuka (UG106), Namusoga (UG111), NN (UG112), Bunduguz Empyaka 2 (UG114), Dimbuka (UG116), New Kawogo (UG117), Bitambi (UG118), Sowola 389A (UG119), NN (UG120), NASPOT 11 (UG124), NN (UG127), NN (UG130), NN (UG131), NN (UG132), Kawogo Old (UG135), NN (UG136), Woluganda (UG137), Tanzania (UG142), Malagalia (UG144), Mbale (UG145), TIS-9101 (NG02), CC SPK004 (CIP441768), and Resisto (UG)
Group B, Cluster II	62	K-118 (KE09), Oguroiwe (KE11), SPK004 (KE19), Ubuogo (KE21), Carrot Dar (TZ01), Mayai (TZ02), Carrot C (TZ03), Ukerewe (TZ04), NN (UG06), Tororo 3 (UG23), Kala (UG40), Abuket 1 (UG41), Ejumula (UG43), Kamamanzi (UG44), Opaade (UG52), Anyumel (UG55) and Dares-Salaam Carrot (UG88)‡, Oleke (UG56), NK318L (UG60), Silk Omuyaka (UG65), Liralira (UG68), NN (UG71), Kibanda (UG73), Dimbuka Obuleku (UG76),), Silk (UG78), Otada (UG79), NASPOT 1 (UG80), NASPOT 5 (UG81), NASPOT 5/58 (UG83), Kampala Red (UG84), Silk (UG85, NN (UG87), Suwedi (UG89), NN (UG90), Duduma 2 (UG93), Dduka Enzala (UG95), NN (UG98), NN (UG99) and Mpaifumbiro (UG140)‡, NN (UG100) and NN (UG107)‡, Oketodede (UG101), Nylon (UG102), NN (UG103), Mugiga (UG108), Bunduguza 2 (UG109), Kigaire (UG110), Gulu (UG115), NASPOT 7 (UG121), NASPOT 9 O (UG122), NASPOT 10 O (UG123), NK259L (UG125) and Mukoma (UG128)‡, Silimu (UG129), Dimbuka Bukulula (UG133), Dagadaga (UG134), BND145L (UG138), Tengerere (UG139), Mpambire (UG141), Silk (UG143, Tanzania (UG146), and Excel (CIP440016)
Unclear	4	CC Naveto (CIP440131), Bunduguza Empyaka (UG75), Uganda Mali (UG104), and WT-237 (n.a.§)
Exotic material Cluster I	18	Rwabuganda (UG58), Kyabafuluki (UG82), TIS-9265 (NG01), Rainha (MZ01), Santo Amaro (CIP400011), Dagga (CIP199062.1), Huarmeyano (CIP420020), DLP3163 (CIP420269), Zapallo (CIP420027), Tainung 64 (CIP440189), Beauregard (CIP440132), Caromex (CIP440136), Jewel (CIP440031), W-115 (CIP440424), CC Yanshu 1 (440024), CC Xushu 18 (440025), CC Jonathan (CIP420014), and CC Resisto (CIP440001)
Total	141	

† Koromojo Red (UG96 and UG113) with Jaccard’s similarity coefficient of 1 (underlined) is truly identical because it was used twice in the crossing block and labeled with different codes, so that the number of clones in Group A is 56.

‡ Pairs of genotypes with Jaccard’s similarity coefficient of 1 (underlined), but morphologically different in at least one trait description.

§ n.a., not available.

For six pairs of genotypes in Cluster II, the Jaccard coefficient was 1.0 (Fig. 2, Table 4, Supplemental Table 2). The pair ‘Koromojo Red’ (UG96 and UG113) was truly identical because the cultivar was used twice in the crossing block. For the other clone pairs with Jaccard coefficients of 1.0, it remained unclear whether they were truly identical because they were described differently in at least one morphological trait or were not amplified for one primer (Supplemental Tables S1 and S2).

## DISCUSSION

With the objective to test the suitability of HEBS for sweetpotato breeding in SSA, gene pools must be established. It is preferable that these pools be mainly formed by local material. Sweetpotato gene pools have so far only been established at CIP in Peru (Grüneberg et al., 2015). However, increasing genetic gains in SSA has priority at CIP. There are first steps of using the independent breeding nurseries in southern and northern Mozambique, as well as those in southern and northern Ghana, as separate gene pools, which can be considered as a preliminary gene pool separation. However, for the large polycross seed nursery at Namulonge in Uganda, there is no information that would allow conducting such a preliminary separation of parental material.

Some breeders argue that recombining hexaploid sweetpotato in polycrosses creates a huge amount of genetic variation but that population means for yield and SPVD resistance do not greatly improve. Indeed, there appears to have been almost no improvement in SPVD resistance. Typically, <0.2% of 1000 clones are resistant to SPVD in breeding populations at Namulonge, as was observed almost two decades ago (Mwanga et al., 2002a, 2002b). A HEBS using reciprocal recurrent selection could be attractive for sweetpotato breeders, especially in EA where SPVD pressure is extremely high, because enhanced inbreeding within gene pools is speculated to lead to higher genetic gains for SPVD resistance. Resistance to SPVD is thought to be recessively inherited involving one or two genes (Mwanga et al., 2002a, 2002b) and the frequency of recessive homozygous loci in a hexaploid population is extremely low (Gallais, 2003). Inbreeding in gene pools, in association with recombination among gene pools to avoid sacrificing heterozygosity, could be a solution.

Our hypothesis that the large number of parents at the breeding platform at Namulonge (135 polycross parents) can be divided into groups was confirmed. As expected, the 10 exotic parents formed a cluster at high aggregation level (Cluster I, Fig. 2; exotic material, Table 4). The parents of African origin formed a separate cluster (Cluster II, Fig. 2) at an aggregation level only slightly lower than the aggregation level comprising exotic parental material and CCs of non-African origin. This African Cluster II was further divided in two groups and gene pools, respectively (Groups A and B, Fig. 1; Groups A and B, Table 4). The aggregation level compared with Cluster I (Fig. 2) indicated a large genetic diversity within Groups A and B of Cluster II, respectively. With respect to genetic diversity, there appears to be little need to introduce non-African clones into the breeding program at Namulonge, and this could even be harmful (e.g., by allowing a gene flow from clones that are highly susceptible to SPVD into EA breeding populations). The cluster pattern observed is consistent with the grouping of CCs (Fig. 2). The five non-African CCs were grouped in Cluster I or in a small independent subcluster, which was neither Cluster I nor II, whereas the CC from Africa (i.e., SPK004 obtained from the CIP genebank) was in Cluster II. SPK004 (CIP441768 held in trust at the CIP genebank) might not be the clone originally thought to be acquisitioned by the genebank. The parent Resisto (UG) at Namulonge appeared to be a local African clone with some striking attributes in common with CC Resisto (CIP440001).

The most striking result of our study was the possibility of proposing for parents of African origin at Namulonge (Cluster II, Fig. 2) two respective groups and gene pools (Table 4), with each pool exhibiting large molecular genetic variation. Distinct groups within adapted germplasm in allogamous crops are raising interest among breeders targeting heterosis and reciprocal recurrent selection schemes. This has been the basis of maize hybrid breeding (Hallauer et al., 2010) and the first step in hybrid breeding for many other crops (Melchinger and Gumber, 1998). To avoid confusion, we propose that in sweetpotato clone breeding the emphasis of gene pool separation and HEBS must be on improved hybrid populations for selection of best clones, and not homogeneous hybrids generated from homozygous inbred lines. However, partially inbred groups or populations in clone breeding could be important in increasing genetic gains for quality and resistance to diseases. A HEBS in sweet-potato breeding comprising A, B, and A ˟ B populations might increase the chances of generating more good A ˟ B crosses with respect to yield and SPVD resistance. The history of maize hybrid breeding (Hallauer et al., 2010) shows that maize breeding gradually entered into classical hybrid breeding from observations made on population hybrids (introgression of Northern Flints and Southern Dents), and the population hybrid variety concept in maize is still worth investigating for certain agriculture sectors (Carena, 2005).

An indication of large genetic diversity in our proposed gene pools (Groups A and B, Fig. 2) was that Groups A and B joined at a Jaccard coefficient of ?0.55 to form Cluster II. The two groups (Gene Pools A and B) in Cluster II (mainly adapted EA parental breeding material) were more distinct after PCoA (Fig. 3). However, DAPC additionally indicated a potential clustering (Clusters 2–4, Fig. 4) within Group A of Cluster II (Fig. 2a). Simulations by Bayesian model-based inference of population structures (Fig. 5) provided further evidence that grouping Cluster II clones (mainly local parental material) into two breeding pools was the most appropriate gene pool separation (Groups A and B).

The parental material in use (135 clones) at Namu-longe comprises mainly local parents (122 clones) and ~10% exotic parents (13 clones), which should be suitable to develop locally adapted breeding material. Using 141 clones, 135 parental clones, six CCs (Table 1), and 31 SSR primers (Table 2), 214 polymorphic alleles were found with an average of 7.13 alleles per primer (Table 3). Compared with the primar y diversity center of sweetpotato (South America, Central America, and the Caribbean), the genetic diversity of the EA breeding platform is indeed large but, as expected, slightly smaller. Roullier et al. (2011) analyzed 329 accessions from South America, Central America, and the Carib-bean and reported 4 to 23 alleles per primer using 13 SSR primers (6 of these 13 primers were also used in our study) Some of the SSR primers used in this study (i.e., Ib-242, Ib-286, and Ib-297) were also used by Veasey et al. (2008) in Brazilian germplasm and resulted in similar numbers of polymorphic alleles per primer to those in our study. The same holds true for a germplasm study in Burkina Faso (Koussao et al., 2014) that used two commonly used primers (IBS144 and IBS199). With respect to EA germplasm (seven primers commonly used), higher numbers of alleles were previously reported for some primers (Tumwegamire et al., 2011b): IbJ116a, Ib-242, IbCIP1, IbJ544b, IbJ522a, IbC12, and Ib-297. This might be because Tumwegamire et al. (2011b) used germplasm from more African countries. The set of primers used in our study was highly informative and suitable for analyzing sweetpotato (Table 3). The average PIC (0.75) was larger than the threshold of PIC ³ 0.50 (Botstein et al., 1980). The values of *D*_L_ for the primer set (average *D*_L_ = 0.82, Table 3) represent the power to differentiate a single genotype from an infinite population (Table 3). We consider that IbN37, Ib-297, IbC12, IBS144, and IBS199 were the most useful primers (*D*_L_ = 0.95–0.97).

The Jaccard coefficients with a mean of 0.542 and range of 0.298 to 1.00 were a clear indicator of high genetic variability among the accessions analyzed. The distribution of similarity agrees with previous reports (Zhang et al., 2001; Yada et al., 2010b; Tumwegamire et al., 2011b). It has been argued that large genetic distances between sweetpotato genotypes can be expected even in smaller populations due to hexaploidy, heterozygosity, self-incompatibility, and ease of sexual seed generation (Grüneberg et al., 2015). Other studies also mention propagation by cloning as a driving factor for new genetic diversity in sweetpotato (He et al., 1995; Zhang and Xie, 1998). The AMOVA for the proposed two gene pools revealed lower but significant variation between parents of Groups A and B (14%) than within groups (86%). The amount of molecular variance among parental material of sugar beet breeding programs is much smaller (2.6%; De Riek et al., 2001). Our observed molecular variance between groups and Gene Pools A and B, respectively, was still larger than in studies for seven tropical maize populations (Reif et al., 2003), with 10.2% between-population variation, and soybean [*Glycine max* (L.) Merr.] landraces from three West Pacific countries with 12.4% variation among countries (Li and Nelson, 2001).

With respect to duplicates among the parental material in use at Namulonge, only one pair of accessions was certainly duplicated (Koromojo Red, UG96, and UG113, Group A of Cluster II in Fig. 2 and Table 4), which reduces the number of parents in the crossing block of Namulonge to 134. Some accessions could be duplicates on the basis of the Jaccard coefficient of one (five pairs indicated in Table 4), but accessions in these pairs were described differently for at least one morphological trait (Supplemental Table S1) or did not amplify for one primer. Currently, we suggest that all five clone pairs remain as parents in the crossing block. With this treatment of the “duplicates” identified in our study, Gene Pools A and B comprised 56 and 62 parents, respectively (Table 4). This number of parents should be sufficient to test whether Gene Pools A and B are mutually heterotic and to discard a larger number of parents (40–50%) in each gene pool on the basis of poor offspring performance concerning yield and SPVD susceptibility. We expect that this effort will result in significant improvement of the breeding population at the East African sweetpotato breeding platform in Namulonge. Moreover, this presents the option to repeat elite cross combinations on a large scale for National Agriculture Research System partners. A remaining critical issue is how exotic and unclearly grouped parents (17 clones excluding CCs, Table 4) should be treated in the breeding platform at Namulonge. Genetic diversity in the proposed Gene Pools A and B is certainly large. The exotic and unclearly grouped parents might not be needed. Only in cases for which breeders consider that the parents have valuable attributes not present as alleles in Gene Pools A and B should the exotic material be kept apart to establish a separate prebreeding program with local EA parents.

Our study showed a successful separation of gene pools in a large set of parental sweetpotato material using SSR markers. Further studies are required to test the hypothesis that the gene pools are mutually heterotic. Our study should help in the search for gene pools in other clonally propagated crops for testing of HEBS.

## Supplemental Material

Supplemental tables for this article are available online. Tables include a description of clones used for the genetic diversity study (parental material and checks; Supplemental Table S1), and specific groupings of parental material based on Jaccard’s SSR and UPGMA (Supple-mental Table S2).
