# Supplementary material for: Gene Pool Subdivision of East African Sweetpotato Parental Material
Source: Crop Sci. 2018 Sep 6;58(6):2302–14. doi: 10.2135/cropsci2017.11.0695 (PMC7680937; doi:10.2135/cropsci2017.11.0695)
Supplement: Supplementary file 1 [file CROPSCI-58-06-2302-s001.pdf]

Supplemental Material

Gene pool subdivision of East African sweetpotato parental material

Maria C. David, Federico C. Diaz, Robert O.M. Mwanga, Silver Tumwegamire, Roberto C. Mansilla and Wolfgang J. Grüneberg.

Contents

Supplemental Table S1 ....

Supplemental Table S2. ....

Supplemental Table S1. Description of clones used for the genetic diversity study (parental material and checks).

Clone name	Local code	Clone type[¥]	Country of origin[¶]	Plant type	Flesh color[#]	Skin Color[#]	Shape	CIP code	Uganda germplasm code
**Clones used as parental material**									
K-118	KE09	FV	KEN	Semi-erect	PO[‡]	C	Long elliptic	na	Na
Oguroiwe	KE11	FV	KEN	Semi-erect	C	C	Long elliptic	na	Na
K-566632	KE14	MV	KEN	Semi-erect	O	PR	Round elliptic	na	Na
SPK004	KE19	FV	KEN	Spreading	PO	P	Long irregular	441768	na
Ubuogo	KE21	FV	KEN	NA	C	PR	NA	na	na
Rainha	MZ01	FV	MOZ	NA	PY	PP	NA	na	TOR3
TIS9265	NG01	MV	NGA	Semi-erect	C	C	Obovate	440076	na
TIS9101	NG02	MV	NGA	Semi-erect	O	PR	Long elliptic	440099	na
Mugande	RW01	MV	RWA	NA	Y	C	NA	na	na
Carrot Dar	TZ01	FV	TZA	Semi-erect	DO	C	Long elliptic	na	na
Mayai	TZ02	FV	TZA	Semi-erect	DO	C	Long elliptic	na	na
Carrot-C	TZ03	FV	TZA	Spreading	DO	C	Long irregular	na	KTK001
Ukerewe	TZ04	MV	TZA	Semi-erect	Y	PR	Elliptic	na	na
Magabari	UG05	FV	UGA	NA	C	C	NA	na	KBL172
NN	UG06	FV	UGA	Semi-erect	O	C	Long elliptic	na	KMI61
Karebe	UG15	FV	UGA	Semi-erect	C	C	Elliptic	na	MBR536
Kigabali	UG19	FV	UGA	NA	C	C	NA	na	KBL618
Kyebandula	UG20	FV	UGA	Semi-erect	C	C	Long elliptic	na	MLE163
Tororo 3	UG23	FV	UGA	Semi-erect	C	C	Long elliptic	na	na
Osapat	UG29	FV	UGA	Erect	Y	C	Obovate	na	SRT01
Kala	UG40	FV	UGA	NA	PO[‡]	C	NA	na	KMI88
Abuket 1	UG41	FV	UGA	Semi-erect	O	P	Long elliptic	na	SRT33
Ejumula	UG43	FV	UGA	Spreading	DO	C	Long irregular	443750	na
Kamamanzi	UG44	FV	UGA	NA	C	P	NA	na	KBL619
NN	UG45	FV	UGA	NA	Y	P	NA	na	IGA978
Wagabolige	UG47	FV	UGA	Spreading	C	C	Round	na	PAL149
Osukut	UG51	FV	UGA	NA	PY	C	NA	na	SRT43
Opaade	UG52	FV	UGA	NA	C	P	NA	na	KMI88
Kakoba	UG53	FV	UGA	Semi-erect	Y	PR	Elliptic	na	KSR662
Epura Amojong	UG54	FV	UGA	NA	C	C	NA	na	SRT28
Anyumel	UG55	FV	UGA	Erect	PO[‡]	C	Round elliptic	na	SRT35
Oleke	UG56	FV	UGA	NA	Y	P	NA	na	APA335

Supplemental Table S1. Continued

Clone name	Local code	Clone type[¥]	Country of origin[¶]	Plant type	Flesh color[#]	Skin Color[#]	Form	CIP code	Uganda germplasm code
Kalobo	UG57	FV	UGA	NA	C	NA	NA	na	MSD431
Rwabuganda	UG58	FV	UGA	NA	C	C	NA	na	KRE696
Kibogo	UG59	FV	UGA	NA	C	P	NA	na	KRE723
NK318L	UG60	BL	UGA	NA	C	PR	NA	na	NK318L
NN	UG61	FV	UGA	NA	C	BO	NA	na	APA356
Kyebandira 2	UG62	FV	UGA	NA	C	C	NA	na	RAK848
Anamoyito	UG63	FV	UGA	NA	C	PR	NA	na	LIR302
Mary	UG64	FV	UGA	NA	C	C	NA	na	SRT40
Silk Omuyaka	UG65	FV	UGA	NA	C	C	NA	na	PAL94
Koromojo	UG66	FV	UGA	NA	Y	C	NA	na	ARA209
Tedolo Keren 1	UG67	FV	UGA	NA	C	C	NA	na	LIR258
Liralira	UG68	FV	UGA	NA	C	C	NA	na	APA323
Kahungezi	UG69	FV	UGA	Semi-erect	C	PR	Ovate	na	MBR552
Burundi	UG70	FV	UGA	NA	C	C	NA	na	KBL611
NN	UG71	FV	UGA	NA	Y	C	NA	na	RAK786
Kalebe	UG72	FV	UGA	NA	W	P	NA	na	KRE691
Kibanda	UG73	FV	UGA	NA	Y	C	NA	na	BSH740
NN	UG74	FV	UGA	NA	C	C	NA	na	LUW1230
Bunduguza Empyaka	UG75	FV	UGA	NA	W	Y	NA	na	IGA994
Dimbuka Obuleku	UG76	FV	UGA	NA	W	C	NA	na	MSK1040
Dimbuka	UG77	FV	UGA	NA	C	P	NA	na	MKN171
Silk	UG78	FV	UGA	NA	C	C	NA	na	KML875
Otada	UG79	FV	UGA	NA	C	PR	NA	na	LIR257
NASPOT 1	UG80	MV	UGA	Spreading	Y	C	Ovate	191133.1	NASPOT 1
NASPOT 5	UG81	MV	UGA	Spreading	O	C	Round elliptic	191133.5	NASPOT 5
Kyabafuluki	UG82	FV	UGA	Spreading	C	C	Round elliptic	na	MBR605
NASPOT5/58	UG83	BL	UGA	NA	O	O	NA	na	NASPOT5/58
Kampala Red	UG84	FV	UGA	Semi-erect	C	PR	Long elliptic	na	KMI59
Silk	UG85	FV	UGA	NA	C	C	NA	na	LUW1254
NASPOT 3	UG86	MV	UGA	Spreading	C	C	Ovate	191133.3	NASPOT 3
NN	UG87	FV	UGA	NA	W	C	NA	na	RAK835

Supplemental Table S1. Continued

Clone name	Local code	Clone type¥	Country of origin¶	Plant type	Flesh color#	Skin Color#	Form	CIP code	Uganda germplasm code
Dar-es-Salaam Carrot	UG88	FV	TZA	NA	O	C	NA	na	SRT37
Suwedi	UG89	FV	UGA	NA	Y	PR	NA	na	MSD384
NN	UG90	FV	UGA	NA	W	P	NA	na	KBL648
Tuulansime	UG91	FV	UGA	NA	C	PR	NA	na	MLE191
Bungoma	UG92	FV	UGA	Semi-erect	C	PR	Elliptic	na	MLE199
Duduma 2	UG93	FV	UGA	NA	C	C	NA	na	KML956
NN	UG94	FV	UGA	NA	W	C	NA	na	MPG1122
Dduka Enzala	UG95	FV	UGA	NA	Y	Y	NA	na	RAK808
Koromojo Red	UG96	FV	UGA	NA	C	C	NA	na	ARA224
Bikiramaria	UG97	FV	UGA	NA	Y	C	NA	na	MPG1158
NN	UG98	FV	UGA	NA	W	Y	NA	na	KBL631
NN	UG99	FV	UGA	NA	Y	Y	NA	na	RAK819
NN	UG100	FV	UGA	NA	C	C	NA	na	KML881
Oketodede	UG101	FV	UGA	Semi-erect	C	C	Round elliptic	na	APA352
Nylon	UG102	FV	UGA	NA	W	C	NA	na	LUW1274
NN	UG103	FV	UGA	NA	C	P	NA	na	MKN1168
Uganda Mali	UG104	FV	UGA	NA	Y	P	NA	na	SRT41
NN	UG105	FV	UGA	NA	PP	C	NA	na	KML960
Munafu Dimbuka	UG106	FV	UGA	NA	W	C	NA	na	MSK1079
NN	UG107	FV	UGA	NA	C	C	NA	na	MKN1224
Mugiga	UG108	FV	UGA	NA	C	P	NA	na	BSH741
Bunduguza 2	UG109	FV	UGA	NA	C	C	NA	na	SRT24
Kigaire	UG110	FV	UGA	NA	C	BO	NA	na	SRT27
Namusoga	UG111	FV	UGA	NA	C	P	NA	na	PAL134
NN	UG112	FV	UGA	NA	C	P	NA	na	PAL148
Koromojo Red	UG113	FV	UGA	NA	C	C	NA	na	ARA224
Bunduguza Empyaka 2	UG114	FV	UGA	NA	W	P	NA	na	IGA998
Gulu	UG115	FV	UGA	NA	C	P	NA	na	MPG1148
Dimbuka	UG116	FV	UGA	NA	W	C	NA	na	HMA496
New Kawogo	UG117	FV	UGA	NA	W	PR	NA	441743	MSK1056
Bitambi	UG118	FV	UGA	NA	C	PR	NA	na	MSK1025

Supplemental Table S1. Continued

Clone name	Local code	Clone type[¥]	Country of origin[¶]	Plant type	Flesh color[#]	Skin Color[#]	Form	CIP code	Uganda germplasm code
Sowola (389A)	UG119	FV	UGA	Semi-erect	C	BO[†]	Elliptic	441744	SWL389A
NN	UG120	FV	UGA	NA	Y	P	NA	na	KML942
NASPOT 7	UG121	MV	UGA	Semi-erect	O	PR	Ovate	na	NASPOT 7
NASPOT 9 O	UG122	MV	UGA	Semi-erect	O	PR	Ovate	na	NASPOT 9 O
NASPOT 10 O	UG123	MV	UGA	Semi-erect	O	PR	Long irregular	na	NASPOT 10 O
NASPOT 11	UG124	MV	UGA	NA	C	PR	NA	100201	NASPOT 11
NK259L	UG125	BL	UGA	NA	C	PR	NA	na	NK259L
Semanda	UG126	FV	UGA	NA	C	P	NA	na	MPG1136
NN	UG127	FV	UGA	NA	C	PR	NA	na	MSK1094
Mukoma	UG128	FV	UGA	NA	C	C	NA	na	KMI76
Silimu	UG129	FV	UGA	NA	C	P	NA	na	IGA983
NN	UG130	FV	UGA	NA	C	Y	NA	na	MKN1180
NN	UG131	FV	UGA	NA	W	C	NA	na	MKN1210
NN	UG132	FV	UGA	NA	W	C	NA	na	LUW1257
Dimbuka-Bukulula	UG133	FV	UGA	Semi-erect	C	C	Long irregular	443752	na
Dagadaga	UG134	FV	UGA	NA	C	C	NA	na	PAL108
Kawogo Old	UG135	FV	UGA	Semi-erect	C	BO[†]	Ovate	na	HMA490
NN	UG136	FV	UGA	NA	C	C	NA	na	KBL650
Woluganda	UG137	FV	UGA	NA	C	C	NA	na	MLE179
BND145L	UG138	BL	UGA	NA	C	C	NA	na	BND145L
Tengerere	UG139	FV	UGA	NA	C	C	NA	na	PAL133
Mpaifumbiro	UG140	FV	UGA	NA	C	C	NA	na	KML875
Mpambire	UG141	FV	UGA	NA	C	Y	NA	na	MPG1146
Tanzania	UG142	FV	UGA	NA	C	P	NA	na	ARA236
Silk	UG143	FV	UGA	NA	C	C	NA	na	LUW1254
Malagalia	UG144	FV	UGA	NA	C	PR	NA	na	MSD380
Mbale	UG145	FV	UGA	NA	Y	C	NA	na	MPG1151
Tanzania	UG146	FV	UGA	Spreading	Y	C	Long elliptic	440166	HMA493
**Non-African clones used as parental material**									
Dagga	NA	MV	PER	Semi-erect	Y	P	Ovate	199062.1	na
Beauregard	NA	MV	USA	Spreading	O	R	Long elliptic	440132	na
Caromex	NA	MV	USA	Spreading	O	BO	Obovate	440136	na
DLP3163	NA	FV	PER	Spreading	Y	C	Elliptic	420269	na

Supplemental Table S1. Continued

Clone name	Local code	Clone type¥	Country of origin¶	Plant type	Flesh color#	Skin Color#	Shape	CIP code	Uganda germplasm code
Excel	NA	MV	USA	Spreading	O	O	Long elliptic	440016	Na
Huarmeyano	NA	FV	PER	Extremely spreading	C	O	Round	420020	na
Jewel	NA	MV	USA	Semi-erect	DO	PR	Round elliptic	440031	na
Resisto (UG)	NA	MV	USA	Semi-erect	DO	BOⱡ	Ovate	440001	na
Santo Amaro	NA	MV	BRA	Extremely spreading	O	R	Long elliptic	400011	na
Tainung 64	NA	MV	TWN	Semi-erect	O	O	Long irregular	440189	na
W-115	NA	MV	USA	Spreading	O	NA	Long elliptic	440424	na
WT-237	NA	MV	na	NA	O	BO	NA	na	na
Zapallo	NA	MV	PER	Spreading	O	C	Obovate	420027	na
**Check clones for the diversity study and not used as parental material**									
Jonathan	NA	MV	PER	Spreading	DO	O	Long elliptic	420014	na
Naveto	NA	FV	PNG	Extremely spreading	W	P	Long elliptic	440131	na
Resisto (CIP)	NA	MV	USA	Spreading	DO	O	Round	440001	na
SPK004 (CIP)	NA	FV	KEN	Extremely spreading	C	R	Long elliptic	441768	na
Xushu 18	NA	MV	CHN	Semi-erect	PY	Y	Long elliptic	440025	na
Yanshu 1	NA	MV	CHN	Semi-erect	C	R	Long elliptic	440024	na

¥ BL,breeding line; FV, farmer variety; MV, modern variety.
¶ BRA, Brazil; CHN, China; KEN, Kenia; MZN, Mozambique; NGA, Nigeria; PER, Peru; PNG, Papua New Guinea; RWA, Rwanda; TWN, Taiwan; TZN, Tanzania; UGA, Uganda; USA, United States of America.
# BO, brownish orange; C, cream; DC, dark cream; DO, dark orange; DY, dark yellow; O, orange; P, pink; PO, pale orange; PP, purple; PR, purple red; PY, pale yellow; R, red; W, white; Y, yellow. Trait characteristic is (‡) light orange and (ⱡ) brown for flesh and skin color, respectively, in Tumwegamire et al. (2011).
NA: not available, na: not applicable

Supplemental Table S2. Specific groupings of parental material based on Jaccard's simple sequence repeat (SSR) and unweighted pair group method analysis (UPGMA).

Groups	No of pairs/clones	Clones
Accession pairs joining at Jaccard similarity coefficient of 1	6 pairs (11 clones; UG96 and UG113 are the same genotype)	Koromojo Red (UG96) and Koromojo Red (UG113), Bikiramaria (UG97)[§] and Semanda (UG126)[§], NK259L (UG125)[§§] and Mukoma (UG128)[§§], Anyumel (UG55)[¶] and Dar-es-Salaam Carrot (UG88)[¶], NN (UG100)[¶¶] and NN (UG107)[¶¶], and RAK819 (UG99)[‡] and Mpaifumbiro (UG140)[‡].
Accession pairs in group A of cluster II joining at similarity larger than 0.90 and smaller than 1	9 pairs	TIS-9101 (NG02) and NASPOT 3 (UG86), MPG1122 (UG94) and MSK1079 / Dimbuka (UG106), UG142 (ARA236 / Tanzania) and SPK004 (CC), UG20 (MLE163 / Kyebandula) and UG05 (KBL172 / Magabari), UG72 (KRE691 / Kalebe) and UG15 (MBR536 / Karebe), UG136 (KBL650) and UG63 (LIR302 / Anamoyito), UG64 (SRT40 / Mary) and UG120 (KML942), UG54 (SRT28 / Epura Amojong) and UG92 (MLE199 Bungoma), and UG66 (ARA209 Koromojo) and UG116 (HMA496 Dimbuka)].
Accession pairs in group B of cluster II joining at similarity larger than 0.90 and smaller than 1	11 pairs	Dimbuka Obuleku (UG76) and Dimbuka-Bukuluka (UG133), Silk (UG85) and Silk (UG143), Silimu (UG129) and Dagadaga (UG134), NASPOT_9_O (UG122) and NASPOT_10_O (UG123), Oleke (UG56) and Liralira (UG68), Kampala Red (UG84) and MKN1168 (UG103), Dduka Enzala (UG95) and Mpambire (UG141), KBL648 (UG90) and Duduma 2 (UG93), Kala (UG40) and Tanzania (UG146), Silk Omuyaka (UG65) and Tengerere (UG139), and Ejumula (UG43) and Carrot_C (TZ03)
OFSP FVs from Africa found in group A of cluster II	2 clones	SPK004 (CIP)[†] and Resisto (UG)[‡].
OFSP FVs from Africa found in group B of cluster II	11 clones	K-118 (KE09), SPK004 (KE19), Carrot_D (TZ01), Mayai (TZ02), Carrot_C (TZ03), UG06, Kala (UG40), Abuket_1 (UG41), Ejumula (UG43), Anyumel (UG55), and Dar_es_Salaam_Carrot (UG88).

[§] Bikiramaria (UG97, yellow flesh root) and Semanada (UG126, cream flesh root).
[§§] NK259L (UG125) is a BL from Uganda and Mukoma (UG128) is a FV collected in Mukono / Uganda.
[¶] Anyumel (UG55) and Dar-es-Salaam Carrot (UG88) differ in intensity of orange flesh color (UG88 was not amplifying for primer IbY53).
[¶¶] NN (UG100) has yellow secondary root flesh color (not presented) and NN (UG107) has no differ in secondary root flesh color (UG107 was not amplifying for primer IbY46)
[‡] NN (UG99) and Mpaifumbiro (UG140) are different in root shape and collected from a different districts.
[†] from CIP's genebank in Peru and labeled as FV.
[‡] parent in crossing block at Namulonge / Uganda and grouping with African FV (Fig. 2).
